# Cancellation of the more complicated ophthalmic inpatient surgeries in a tertiary general hospital: a 10-year retrospective study

**DOI:** 10.3389/fmed.2024.1406140

**Published:** 2024-08-29

**Authors:** Xinyu Zhao, Qing Zhao, Yanfang Wu, Shiyu Cheng, Chuting Wang, Lihui Meng, Xingwang Gu, Youxin Chen

**Affiliations:** ^1^Department of Ophthalmology, Peking Union Medical College Hospital, Chinese Academy of Medical Sciences, Beijing, China; ^2^Key Laboratory of Ocular Fundus Diseases, Chinese Academy of Medical Sciences & Peking Union Medical College, Beijing, China

**Keywords:** inpatient surgery, surgery cancellation, ophthalmic surgery, incidence, related factor, cause

## Abstract

**Background:**

Although ophthalmic ambulatory surgeries are less costly and may enhance the more efficient utilization of hospital resources, inpatient surgeries were preferable alternatives for patients with complicated eye diseases or poor general health. However, the incidence, causes, and related factors of ophthalmic inpatient surgery cancellation remained largely unknown.

**Methods:**

The electronic medical records of ophthalmic inpatient surgeries between January 2012 and December 2022 was retrospectively reviewed. Cancellation-related factors were explored using multivariate logistic regression analysis and the reasons of cancellation were also evaluated.

**Results:**

In total, 820 cancelled surgeries and 42,073 performed surgeries were included, with a cancellation rate of 1.9%. Any other ocular comorbidities were risk factors for cancellation (odds ratio (OR) 1.872, 95% confidence intervals (CI) 1.504–2.331; *p* < 0.001), while older age (OR 0.990, 95% CI 0.986–0.995; *p* < 0.001), local residence (OR 0.809, 95% CI 0.692–0.947; *p* = 0.008), any systemic comorbidities (OR 0.740, 95% CI 0.616–0.889; *p* = 0.001), and previous history of surgeries (OR 0.403, 95% CI 0.341–0.476; *p* < 0.001) were negatively associated with surgery cancellation. The top two categorical cancellation reasons were medical factors (508, 62.0%) and patient-related factors (285, 34.8%). “Patient/family refused surgery” (127, 15.5%), “acute conjunctivitis or uveitis relapse” (103, 12.6%), and “ocular condition improved and procedure no longer indicated” (71, 8.7%) were the three most common single cancellation reasons.

**Conclusion:**

Any other ocular comorbidities, younger age, no systemic comorbidities, non-local residence, and no past surgical history were related factors for ophthalmic inpatient surgery cancellation. The majority of cancellations were due to patient-related or medical factors. Great importance should be attached to the cancellation of the more complicated inpatient surgeries and further efforts are warranted to explore how to reduce cancellation.

## 1 Introduction

Operating rooms are important workspaces with the extensive cost of human resources. Cancellation of scheduled surgeries would impair the efficient utilization of operating rooms, cause immense waste of manpower, and increase the cost of care (1, 2). Previous studies suggested the revenues lost of around $1,430 to $1,700 per hour due to surgery cancellation (3). Further studies estimated that the average cost per ophthalmic surgery cancellation was around $379 (4). Surgery cancellation would double their medical costs because of the consequent readmission and repetitive preoperative examinations (5). In addition, surgery cancellation may also reduce the patient’s satisfaction with medical care and negatively influence the staff-to-patient relationships (5, 6). Therefore, identifying and then efficiently managing reasons and related factors for surgery cancellation may enhance the efficiency of operating rooms and improve the medical experience of patients.

Recently, surgery cancellation has been largely investigated (7, 8). However, studies specifically focused on ophthalmic surgeries were limited and these existing studies mainly focused on ambulatory surgeries (9, 10). Although ophthalmic ambulatory surgeries are less costly and may lead to more efficient utilization of hospital resources, hospitalisation remains a preferable option for difficult cases and patients with poor or more complicated general health. Inpatient care enables ophthalmologists to adequately prepare patients for surgery and allows for extended postoperative monitoring to ensure the patient’s health condition remains stable. However, the incidence and causes of these more complicated ophthalmic inpatient surgeries cancellation remained largely unknown. Furthermore, no studies, to the best of our knowledge, have explored the related factors for these ophthalmic inpatient surgery cancellations. Discovering their related factors might help ophthalmologists to identify patients with a great possibility to cancel the scheduled surgeries so that timely preventive interventions could be adopted to minimise these cancellations in the clinical practise.

In the present study, we aimed to explore the incidence of the cancellation of the more complicated ophthalmic inpatient surgeries and further investigate their related factors and reasons in our medical centre, hopefully providing valuable references for hospitals to better manage this problem.

## 2 Materials and methods

### 2.1 Study design

In this study, we retrospectively reviewed the electronic medical records (EMR) of the ophthalmic inpatient surgeries between January 2012 and December 2022 in the Department of Ophthalmology at Peking Union Medical College Hospital (PUMCH) in Beijing, China. EMRs of those ambulatory ophthalmic surgeries were not included. This study complied with the Helsinki Declaration and the Ethics Committee of PUMCH (approval number: K3857) has approved the study protocol. Informed consents from patients to participate in the study were waived due to its retrospective property.

### 2.2 Variables

Extracted variables were categorised into patient demographics and surgery-related factors. Patient demographics included age, gender, residence (local or nonlocal), any ocular comorbidities, any systemic comorbidities, hypertension, diabetes mellitus, and previous history of surgeries. “Local residence” means patients living in Beijing, while “non-local residence” refers to patients living in provinces or cities outside of Beijing. Surgery-related factors included the season of the surgery, anaesthesia type (local or general anaesthesia), and indications for surgery. The seasons of the surgery were defined as follows: spring as March to May; summer as June to August; autumn as September to November; and winter as December to February. The indications for surgery were classified into 7 different groups, including fundus diseases, cataracts, glaucoma, corneal diseases, strabismus, oculoplastic surgery, and miscellaneous surgeries.

Surgery cancellation was defined as the condition that the surgery has been entered into the surgery scheduling system with all required medical resources (including operating rooms and workforces) well prepared but was then cancelled due to certain reasons. Reasons for surgery cancellation were categorised as (1) patient-related factors (e.g., patient/family refused surgery; patients want to postpone surgery; patients intolerant to the surgery, etc.); (2) medical factors (e.g., change in patient’s systemic or ocular medical status, inadequate preoperative assessments, etc.); (3) surgeon-related factors (e.g., surgeon sick, surgeon unavailable due to personal reasons, etc.); (4) administration-related factors (e.g., intraocular lens unavailable, operating room unavailable, or equipment malfunction); (5) financial factors (e.g., no insurance coverage or economic concerns); (6) no reasons given on EMR. For cancelled cases, readmission for the same underlying issue and the readmission time interval were also retrieved.

### 2.3 Data analysis

Categorical data were displayed as frequency (percentages), and numerical data were presented as mean (standard deviation, SD). The univariable and multivariable logistic regression analyses were used to explore the association of patient demographics and surgery-related factors with surgical cancellation, shown as odds ratio (OR) with 95% confidence intervals (CI). All statistical analyses were performed using the Statistical Packages for the Social Sciences (SPSS) software, version 23.0 (SPSS Inc., Chicago, IL, USA), with two-tailed *p* < 0.05 regarded as statistically significant.

## 3 Results

### 3.1 Incidence of inpatient surgery cancellation

**Figure 1:**
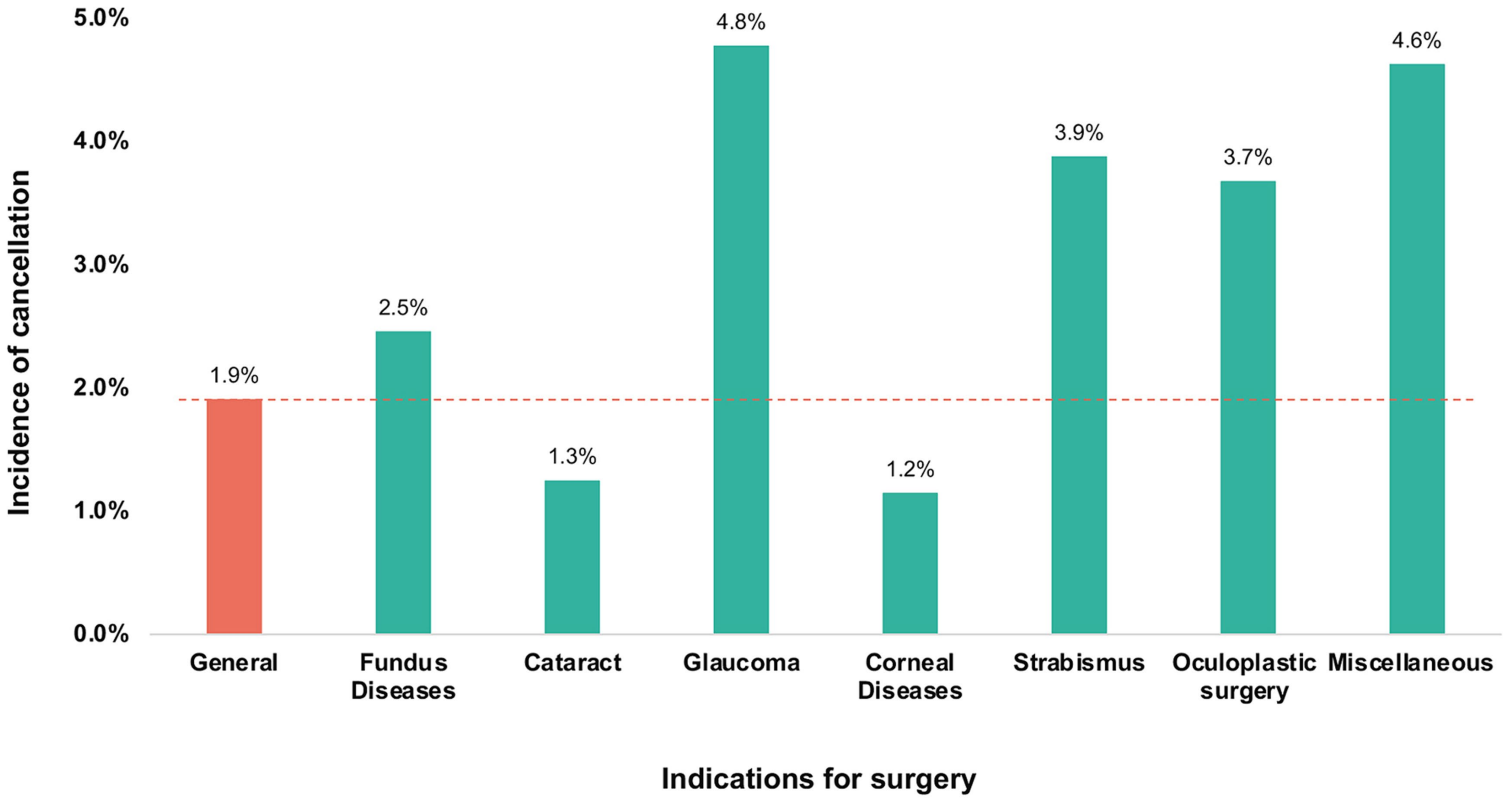
Cancellations of inpatient ophthalmic surgeries in patients with various indications of surgery.

A total of 42,893 scheduled surgeries were included and reviewed. Out of these, 42,073 surgeries were performed successfully and 820 surgeries were cancelled, resulting in a cancellation rate of 1.9%. Amongst the various indications for surgery, glaucoma surgery had the highest cancellation rate of 4.8%, followed by miscellaneous surgeries (4.6%), strabismus surgery (3.9%), and oculoplastic surgery (3.7%) (see Figure 1).

### 3.2 Patient demographics and surgery-related factors

In the cancellation group, the mean age was 51.46 (22.92) years, which was significantly lower than the surgery group [58.31 (18.99); *p* < 0.001]. The cancellation group had a significantly higher percentage of male patients compared to the surgery group (*p* = 0.035). Additionally, the cancellation group had a lower proportion of patients with local residence (*p* = 0.033), a higher incidence of any other ocular comorbidities (*p* < 0.001), a lower incidence of hypertension (*p* = 0.009), and a lower incidence of any systemic comorbidities (*p* < 0.001). Amongst the patients in the cancellation group, 207 (25.2%) had a previous history of surgeries, which was significantly lower than the surgery group [22,361 (53.1%); *p* < 0.001]. The percentage of patients with comorbid diabetes mellitus was similar in both groups (*p* = 0.769) (see Table 1).

**Table 1:** Patient demographics and surgery-related factors for surgery cancellation.

	Cancellation	Surgery	OR (95% CI)	*p*
Number	820	42,073		
Age, years, mean (SD)	51.46 (22.92)	58.31 (18.99)	0.984 (0.981–0.987)	< 0.001^a^
Gender, no. (%)				
Male	408 (49.8%)	19,377 (46.1%)	1.160 (1.010–1.332)	0.035^a^
Female	412 (50.2%)	22,696 (53.9%)		
Residence, no. (%)				
Local	337 (41.1%)	5,841 (44.9%)	0.856 (0.741–0.987)	0.033^a^
Nonlocal	483 (58.9%)	7,162 (55.1%)		
Any other ocular comorbidities, no. (%)	717 (87.4%)	33,477 (79.6%)	1.787 (1.452–2.200)	< 0.001^a^
Systemic comorbidities, no. (%)				
Hypertension	254 (31.0%)	14,877 (35.4%)	0.820 (0.707–0.953)	0.009^a^
Diabetes mellitus	225 (27.4%)	11,740 (27.9%)	0.977 (0.837–1.141)	0.769
Any	475 (57.9%)	27,223 (64.7%)	0.751 (0.653–0.864)	< 0.001^a^
History of surgeries, no. (%)	207 (25.2%)	22,361 (53.1%)	0.298 (0.254–0.349)	< 0.001^a^
The season of the surgery, no. (%)				
Spring	212 (25.9%)	10,908 (25.9%)	0.996 (0.851–1.167)	0.962
Summer	228 (27.8%)	10,993 (26.1%)	1.089 (0.933–1.271)	0.279
Autumn	209 (25.4%)	11,041 (26.2%)	0.961 (0.820–1.127)	0.627
Winter	171 (20.9%)	9,131 (21.7%)	0.951 (0.802–1.127)	0.559
Anaesthesia type, no. (%)				
Local anaesthesia	772 (94.1%)	39,206 (93.5%)	1.125 (0.838–1.509)	0.433
General anaesthesia	48 (5.9%)	2,742 (6.5%)		
Indications for surgery, no. (%)				
Fundus diseases	361 (44.0%)	14,330 (34.1%)	1.523 (1.325–1.750)	< 0.001^a^
Cataract	302 (36.8%)	23,949 (56.9%)	0.441 (0.382–0.509)	< 0.001^a^
Glaucoma	38 (4.6%)	757 (1.8%)	2.674 (1.916–3.732)	< 0.001^a^
Corneal diseases	1 (0.1%)	86 (0.2%)	0.596 (0.083–4.285)	0.607
Strabismus	79 (9.6%)	1,958 (4.7%)	2.184 (1.724–2.767)	< 0.001^a^
Oculoplastic surgery	34 (4.1%)	890 (2.1%)	1.999 (1.409–2.836)	< 0.001^a^
Miscellaneous	5 (0.6%)	103 (0.2%)	2.500 (1.016–6.150)	0.046^a^

^ap < 0.05.^

CI, confidence intervals; OR, odds ratio; SD, standard deviation.

Regarding surgery-related factors, the distribution of seasons for surgery and types of anaesthesia was similar between the two groups (*p* > 0.05). In terms of the indications for surgery, the cancellation group had a significantly higher percentage of patients with fundus diseases (*p* < 0.001), glaucoma (*p* < 0.001), strabismus (*p* < 0.001), oculoplastic surgery (*p* < 0.001), and miscellaneous surgeries (*p* = 0.046). Conversely, the percentage of cataract surgeries was significantly lower in the cancellation group (*p* < 0.001). The percentage of corneal diseases was similar in the cancellation and surgery groups (*p* = 0.607) (see Table 1).

### 3.3 Multivariable logistic regression analysis

The results of the multivariable logistic regression analysis confirmed that the presence of any other ocular comorbidities was a significant risk factor for surgery cancellation (OR 1.872, 95% CI 1.504, 2.331; *p* < 0.001). On the other hand, older age (OR 0.990, 95% CI 0.986, 0.995; *p* < 0.001), local residence (OR 0.809, 95% CI 0.692, 0.947; *p* = 0.008), the presence of any systemic comorbidities (OR 0.740, 95% CI 0.616, 0.889; *p* = 0.001), and a previous history of surgeries (OR 0.403, 95% CI 0.341, 0.476; *p* < 0.001) were found to be negatively associated with surgery cancellation (see Table 2).

**Table 2:** Multivariable logistic regression analysis of potential related factors for surgery cancellation.

	Cancellation	Surgery	OR (95% CI)	*p*
Number	820	42,073		
Age, years, mean (SD)	51.46 (22.92)	58.31 (18.99)	0.990 (0.986–0.995)	< 0.001^a^
Male, no. (%)	408 (49.8%)	19,377 (46.1%)	1.014 (0.877–1.173)	0.854
Local residence, no. (%)	337 (41.1%)	5,841 (44.9%)	0.809 (0.692–0.947)	0.008^a^
Any other ocular comorbidities, no. (%)	717 (87.4%)	33,477 (79.6%)	1.872 (1.504–2.331)	< 0.001^a^
Hypertension, no. (%)	254 (31.0%)	14,877 (35.4%)	0.947 (0.780–1.149)	0.578
Any systemic comorbidities, no. (%)	475 (57.9%)	27,223 (64.7%)	0.740 (0.616–0.889)	0.001^a^
History of surgeries, no. (%)	207 (25.2%)	22,361 (53.1%)	0.403 (0.341–0.476)	< 0.001^a^
Indications for surgery, no. (%)				
Fundus diseases	361 (44.0%)	14,330 (34.1%)	2.498 (0.424–14.720)	0.312
Cataract	302 (36.8%)	23,949 (56.9%)	0.827 (0.140–4.889)	0.834
Glaucoma	38 (4.6%)	757 (1.8%)	4.073 (0.671–24.743)	0.127
Strabismus	79 (9.6%)	1,958 (4.7%)	2.537 (0.425–15.144)	0.307
Oculoplastic surgery	34 (4.1%)	890 (2.1%)	4.360 (0.720–26.407)	0.109
Miscellaneous	5 (0.6%)	103 (0.2%)	3.223 (0.435–23.852)	0.252

^ap < 0.05.^

CI, confidence intervals; OR, odds ratio; SD, standard deviation.

### 3.4 Reasons for inpatient surgery cancellation

Out of the 820 cancelled surgeries, the most common major categorical reason for cancellation was medical factors, accounting for 508 cases (62.0%). Patient-related factors were the second most common reason (285 cases, 34.8%), followed by administration-related factors (7 cases, 0.9%), surgeon-related factors (3 cases, 0.4%), and financial factors (3 cases, 0.4%). Additionally, there were 14 cases (1.7%) where no reasons were provided in the EMR. The most frequently reported single reason for surgery cancellation was “patient/family refused surgery” (127 cases, 15.5%), followed by “the relapse of acute conjunctivitis or uveitis” (103 cases, 12.6%), “ocular condition improved and procedure no longer indicated” (71 cases, 8.7%), and “patient want to postpone surgery” (70 cases, 8.5%) (see Table 3). Amongst all the cancelled cases, 392 patients (47.8%) were readmitted to the hospital for the same underlying issue, with an average time interval of 120 (290) days [range: 1–2,471].

**Table 3:** The reasons for surgery cancellation.

	No. (%)
Patient-related factors	285 (34.8%)
Patient/family refused surgery	127 (15.5%)
Patients want to postpone surgery	70 (8.5%)
Patients intolerant to the surgery	83 (10.1%)
Intolerance to the local anaesthesia	40 (4.9%)
Inability to maintain the surgical position	7 (0.9%)
Claustrophobia onset	36 (4.4%)
Patients from medium/high-risk COVID-19 pandemic regions	4 (0.5%)
Hospital rule violation	1 (0.1%)
Medical factors	508 (62.0%)
Change in patient’s systemic medical status	254 (31.0%)
Upper respiratory tract infection	30 (3.7%)
Skin infection	8 (1.0%)
Fever	31 (3.8%)
Uncontrolled hypertension	34 (4.1%)
Uncontrolled hyperglycemia	28 (3.4%)
Cardiac or cerebrovascular events	45 (5.5%)
Delirium	3 (0.4%)
In menses	3 (0.4%)
In pregnancy	3 (0.4%)
Pancytopenia/neutropenia/thrombocytopenia	7 (0.9%)
Coagulopathy	10 (1.2%)
Hyperkalemia	5 (0.6%)
Other diseases	47 (5.7%)
Change in patient’s ocular medical status	231 (28.2%)
Condition improved and procedure no longer indicated	71 (8.7%)
Acute conjunctivitis or uveitis relapse	103 (12.6%)
Change plan in treatment plan	56 (6.8%)
Lacrimal obstruction	1 (0.1%)
Inadequate preoperative assessments	9 (1.1%)
Incomplete examinations	8 (1.0%)
Nil per os violations	1 (0.1%)
Intraoperative abnormalities	2 (0.2%)
Complications after anaesthesia	12 (1.5%)
Surgeon-related factors	3 (0.4%)
Surgeon sick	2 (0.2%)
Surgeon from medium/high-risk COVID-19 pandemic regions	1 (0.1%)
Surgeon unavailable due to personal reasons	0 (0.0%)
Administration-related factors	7 (0.9%)
Intraocular lens unavailable	3 (0.4%)
Operating room unavailable	3 (0.4%)
Equipment Malfunction	1 (0.1%)
Financial factors	3 (0.4%)
No insurance coverage	1 (0.1%)
Economic concerns	2 (0.2%)
No reasons given on EMR	14 (1.7%)
Total	820 (100.0%)

## 4 Discussion

To the best of our knowledge, this study is the first and largest to explore the incidence, causes, and related factors of the cancellation of the more complicated ophthalmic inpatient surgeries. The cancellation rate in this study was 1.9%. Any other ocular comorbidities were risk factors for surgery cancellation, while older age, local residence, any systemic comorbidities, and past surgical history were negatively associated with the ophthalmic inpatient surgery cancellation. The most common categorical cancellation reasons were medical factors, followed by patient-related factors. The top three single cancellation reasons were “patient/family refused surgery,” “acute conjunctivitis or uveitis relapse,” and “ocular condition improved and procedure no longer indicated.”

The previously reported cancellation rate of ophthalmic ambulatory surgeries varied from 5.3 to 29.5% (11, 12), depending on the medical centre location (e.g., rural or urban), various categories of the performed ophthalmic surgeries, and the presence or absence of official reminders. Cho et al. (13) reported a lower cancellation rate of 4.3% in ophthalmic inpatient surgeries in a large-scale general hospital in Korea. Although no current consensus existed on the acceptable cut-off value for surgery cancellation rate when defining efficient utilization of operating rooms, less than 5% is generally recommended (14). The overall cancellation rate of the more complicated ophthalmic inpatient surgeries in our study was 1.9%. One possible reason is that the surgeon’s assistant would make telephone calls to confirm the completion of all the pertinent laboratory tests and consultations. Previous studies reported that calling patients several days before the date of the scheduled surgery could more than halve the surgery cancellation rate (15, 16). Additionally, these telephone calls could also increase the patients’ satisfaction scores.

Identifying the potentially modifiable related factors of inpatient surgery cancellation can serve as a basis for preoperative interventions aimed at minimising this risk. The association between age and surgery cancellation has been explored in ophthalmic ambulatory surgeries, however, the findings remained contradictory (11, 17). In our study, we found a significantly younger mean age in the cancellation group than in the surgery group and the multivariable logistic regression analysis confirmed older age as a factor negatively associated with the surgery cancellation. For gender, Feleke et al. (18) found a similar percentage of male and female patients in the cancellation group. However, Cho et al. (13) and McIntosh et al. (19) showed that male patients had a higher cancellation rate, consistent with our findings.

In the present study, we found that the existence of any other ocular comorbidities was the risk factor for surgery cancellation. Besides, we also found that the cancellation of the more complicated ophthalmic inpatient surgeries was associated with absence of systemic comorbidities, non-local residence, and no past surgical history. The fear of the surgery may cause some patients to cancel their scheduled surgeries. The past surgical history may help the patients understand more about the procedure, reduce their fear of the procedure itself, and decrease the surgery cancellation due to the single reason of “patient/family refused surgery.” Similarly, Petrone et al. (20) found that the lack of a history of surgery requiring anaesthesia contributed to an increased risk of elective orthopaedic surgery cancellation. Further studies were warranted to validate these associations.

The most frequent single reason for surgery cancellation in our study was “patient/family refused surgery” involving 15.49% of patients, different from the previously reported “absence of the patient on the day of surgery” in the cancellation of ophthalmic ambulatory surgeries (17, 21). This inconsistency possibly results from the different categories of the included ophthalmic surgeries: inpatient surgery versus ambulatory surgery. First, the ambulatory surgeries are typically less complex and carry a lower risk for complications compared to surgical procedures that require hospitalisation. Secondly, in the case of ambulatory surgeries, informed consent is obtained in advance during a preoperative visit to the outpatient clinic, during which time the healthcare provider would explain the surgery-related risks and benefits and address any questions or concerns the patient may have. In inpatient surgeries, patients were admitted 1 day before the surgery date so that they would have enough time to complete the preoperative assessments and receive preoperative treatments. The surgeries would be added to the surgery scheduling system only after the patients were admitted to the hospital. When reviewing EMR, we found that most patients refused the scheduled surgery because they were overly concerned about the possible postoperative surgery-related adverse events and unsatisfying outcomes. For these patients, informing them of the detailed surgery-related risks and complications in the outpatient setting before their admission instead of after hospitalisation might help reduce the cancellation due to “patient/family refused surgery.” However, there can be variations in inpatient surgery consent practises amongst various countries, and it’s important to acknowledge those differences. Informed consents are obtained after the patient’s hospitalisation in our medical centre, rather than during a preoperative visit to the outpatient clinic, which is the practise in the United States or England.

Henderson et al. (11) reported that “the patient ate or drank before the scheduled procedure,” indicating nil *per os* (NPO) violations, as one of the most common preventable causes of ambulatory ocular surgery cancellation in the United States, with an incidence of 31.9% (43/135). In contrast, the incidence of NPO violations in our study was only 0.1% (1/820). This discrepancy may be due to the different settings of ophthalmic surgeries, specifically inpatient versus ambulatory. NPO violations might occur because of forgetfulness amongst elderly patients or a lack of effective reminders the evening before or the morning of surgery. However, in inpatient ophthalmic surgeries, patients are admitted 1 day before the surgery date and receive their first preoperative instruction at admission, including NPO rules. Additionally, both nurses and doctors remind patients not to eat or drink during the NPO period on the evening before and the morning of surgery. These effective reminders likely help reduce the incidence of NPO violations.

Acute infective conjunctivitis, due to viral and/or bacterial infection, is a common problem in the primary care setting (22). It’s usually self-limiting with innocuous clinical findings, with most patients getting better regardless of antibiotic therapy (23, 24). Although acute infective conjunctivitis was regarded as a mild condition, its presence was considered one of the contraindications for ophthalmic surgeries. In our department, patients planned to undergo ophthalmic surgeries were required to prophylactically use levofloxacin eyedrops for 3 days before the date of the surgery. However, we still found that “acute conjunctivitis or uveitis relapse” was the second most common single reason for surgery cancellation, involving 12.56% of patients. The main differential diagnosis for acute infective conjunctivitis is allergic conjunctivitis, especially due to the application of compound tropicamide eye drops. Therefore, when facing preoperative acute conjunctivitis, ophthalmologists should differentiate allergic conjunctivitis because it’s effective to stop the application of tropicamide, with no need for cancelling the scheduled surgery.

In our institution, patients needed to wait for as long as several months to have the chance to undergo ophthalmic inpatient surgeries. In this case, if the long-waited surgeries were cancelled, patients had to wait for another several months to have the rescheduled surgeries. The average time interval between the ophthalmic inpatient surgeries cancellation and readmission found in our study was 120 days. Tewfik et al. (25) also reported substantial delays for the rescheduled surgeries. Also, other patients on the waiting list for surgery would miss the chance to have their surgeries earlier. Except for these time losses, financial losses should also be noted. Individual patients needed to change their work schedule, take unpaid leave from work, and sometimes travel a long distance to undergo surgeries, leading to individual financial costs (26). Mehran et al. (27) estimated that in a tertiary eye hospital in a metropolitan area, the annual lost reimbursement resulting from glaucoma surgery cancellation was $208,306 in total and approximating $3,372 per procedure. Appropriate methods should be explored to minimise unnecessary late cancellations.

To reduce the surgery cancellation rate, Wongtangman et al. (28) recently presented a preoperative prediction tool containing 29 characteristic factors to identify patients at high risk for 24-h surgery cancellation in the Montefiore Medical Centre. Furthermore, previous studies have proposed an outpatient-based preoperative clinic (POC) consisting of multidisciplinary professionals (e.g., anesthesiologists, pharmacists, and nurses) to evaluate patients before hospitalisation (29). However, the effectiveness of this system remained controversial, with various studies reporting its effectiveness (30), partial effectiveness (31), and ineffectiveness (32, 33) in reducing cancellations. Such discrepancies might be explained by the differences in medical centres, patient characteristics, surgery types, and detailed reasons for surgery cancellation. Another factor that healthcare organisations should take into consideration when evaluating the implementation of POC in healthcare settings is their economic implications. Studies that have found POC to be effective in reducing surgery cancellations considered them to be highly cost-effective, because a 1% decrease in cancellation rates can result in revenue savings of approximately $5.6 million at a large medical centre (34). However, it is important to note that establishing an additional preoperative clinic may potentially increase medical costs for patients. Therefore, the overall cost-effectiveness of POC, especially from the patients’ perspective, requires further exploration and evaluation.

Our medical centre serves as the national centre for diagnosing and treating complex and critical diseases, and patients with eye conditions in our hospital are often difficult cases or those with poor or more complicated general health. In such cases, ophthalmic inpatient surgeries are a better option than ambulatory surgeries, which may be more suitable for less complex cases, such as cataract surgeries without any systemic comorbidities. In this study, we aimed to gain insight into the cancellation of more complicated ophthalmic inpatient surgeries, with the hope of providing valuable references for hospitals to better manage this issue. However, we acknowledged the existence of several limitations in this study. First, due to its single-center property, the findings may not be directly generalised to other medical centers. The differences between practise patterns across various healthcare or reimbursement contexts should be taken into consideration when interpreting our findings. Second, 1.7% of the surgery cancellation cases had no documentation of their cancellation reasons given on EMR. Maintaining the integrity and accuracy of EMR is crucial to explore reasons for cancellation and then further propose appropriate solutions. Third, as a retrospective cross-sectional study, the causal relationships between surgery cancellations and related factors cannot be confirmed in this study.

In conclusion, any other ocular comorbidities, younger age, no systemic comorbidities, non-local residence, and no past surgical history were related factors for ophthalmic inpatient surgery cancellation. The majority of cancellations were due to patient-related or medical factors. Great importance should be attached to the cancellation of the more complicated inpatient surgeries and further efforts are warranted to explore how to reduce cancellation.

## Data Availability

The original contributions presented in the study are included in the article/supplementary material, further inquiries can be directed to the corresponding author.

## Abbreviations

CI: confidence intervals
EMR: electronic medical records
OR: odds ratio
POC: preoperative clinic

## References

[1] Shivakumar G Lokesh V. Reasons and appropriate measures to circumvent cancellation of elective surgical cases: a clinical audit of a government teaching hospital. IJMA. (2021) 4:06–10. doi: 10.33545/26643766.2021.v4.i1a.187

[2] Shaheen I Abbass A Elkheir I Arbab S Bur A Geregandi T. Cancellation of elective surgical operations in a teaching hospital at Khartoum Bahri, Sudan. Sudan Med Mon. (2016) 11:45. doi: 10.4103/1858-5000.185230

[3] Dexter F Marcon E Epstein RH Ledolter J. Validation of statistical methods to compare cancellation rates on the day of surgery. Anesth Analg. (2005) 101:465–73. doi: 10.1213/01.ANE.0000154536.34258.A8, PMID: 16037163

[4] Maimaiti N Rahimi A Aghaie LA. Economic impact of surgery cancellation in a general hospital. Ethiop J Health Dev. (2016) 30:94–8.

[5] Armoeyan M Aarabi A Akbari L. The effects of surgery cancellation on patients, families, and staff: a prospective cross-sectional study. J Perianesth Nurs. (2021) 36:695–701.e2. doi: 10.1016/j.jopan.2021.02.009, PMID: 34565663

[6] Robb WB O'Sullivan MJ Brannigan AE Bouchier-Hayes DJ. Are elective surgical operations cancelled due to increasing medical admissions? Ir J Med Sci. (2004) 173:129–32. doi: 10.1007/BF03167925, PMID: 15693380

[7] Liu L Ni Y Beck AF Brokamp C Ramphul RC Highfield LD. Understanding pediatric surgery cancellation: geospatial analysis. J Med Internet Res. (2021) 23:e26231. doi: 10.2196/26231, PMID: 34505837 PMC8463951

[8] Bose SK Dasani S Roberts SE Wirtalla C DeMatteo RP Doherty GM. The cost of quarantine: projecting the financial impact of canceled elective surgery on the nation's hospitals. Ann Surg. (2021) 273:844–9. doi: 10.1097/SLA.0000000000004766 33491974

[9] Sarang B Bhandoria G Patil P Gadgil A Bains L Khajanchi M. Assessing the rates and reasons of elective surgical cancellations on the day of surgery: a multicentre study from urban Indian hospitals. World J Surg. (2022) 46:382–90. doi: 10.1007/s00268-021-06364-1, PMID: 34787712 PMC8724145

[10] Koh WX Phelan R Hopman WM Engen D. Cancellation of elective surgery: rates, reasons and effect on patient satisfaction. Can J Surg. (2021) 64:E155–61. doi: 10.1503/cjs.008119, PMID: 33666393 PMC8064262

[11] Henderson BA Naveiras M Butler N Hertzmark E Ferrufino-Ponce Z. Incidence and causes of ocular surgery cancellations in an ambulatory surgical center. J Cataract Refract Surg. (2006) 32:95–102. doi: 10.1016/j.jcrs.2005.11.013, PMID: 16516786

[12] Mayro EL Pizzi LT Hark LA Murchison AP Wisner D Koka A. A proposed intervention to decrease resident-performed cataract surgery cancellation in a tertiary eye care center. Am Health Drug Benefits. (2018) 11:480–7. PMID: 30746019 PMC6322594

[13] Cho HS Lee YS Lee SG Kim JM Kim TH. Reasons for surgery cancellation in a general hospital: a 10-year study. Int J Environ Res Public Health. (2018) 16:7. doi: 10.3390/ijerph16010007, PMID: 30577514 PMC6338898

[14] Kaddoum R Fadlallah R Hitti E El-Jardali F El Eid G. Causes of cancellations on the day of surgery at a tertiary teaching hospital. BMC Health Serv Res. (2016) 16:259. doi: 10.1186/s12913-016-1475-6, PMID: 27412041 PMC4944432

[15] Haufler K Harrington M. Using nurse-to-patient telephone calls to reduce day-of-surgery cancellations. AORN J. (2011) 94:19–26. doi: 10.1016/j.aorn.2010.12.024, PMID: 21722768

[16] O'Riordan B Reynolds IS Hechtl D Lecot FP Arya S Geoghegan J. Short notice cancellations - an insight into Irish surgical waiting lists. Ir J Med Sci. (2022) 192:807–10. doi: 10.1007/s11845-022-03026-6 35641839 PMC9156352

[17] Xi CH Shi DJ Li YH Zhang Y Yue JY Li M. Analysis of the incidence and reasons of the same-day cancellation of non-cataract ophthalmic ambulatory surgery. Zhonghua Yi Xue Za Zhi. (2022) 102:1608–13. doi: 10.3760/cma.j.cn112137-20220212-00293, PMID: 35644963

[18] Feleke MG Chichiabellu TY Ayalew TL. Magnitude and reasons of surgery cancellation among elective surgical cases in Wolaita Sodo University comprehensive specialized hospital, southern Ethiopia, 2021. BMC Surg. (2022) 22:300. doi: 10.1186/s12893-022-01749-y, PMID: 35927654 PMC9354349

[19] McIntosh B Cookson G Jones S. Cancelled surgeries and payment by results in the English National Health Service. J Health Serv Res Policy. (2012) 17:79–86. doi: 10.1258/jhsrp.2011.011053, PMID: 22315466

[20] Petrone B Fakhoury J Matai P Bitterman A Cohn RM Lutsky L. Predicting elective orthopaedic sports medicine surgical cancellations based on patient demographics. Arthrosc Sports Med Rehabil. (2020) 2:e83–9. doi: 10.1016/j.asmr.2019.11.004, PMID: 32368743 PMC7190548

[21] Fernando BS Cannon PS Mohan M. Cancellation of surgical day cases in an ophthalmic Centre. Acta Ophthalmol. (2009) 87:357–8. doi: 10.1111/j.1755-3768.2008.01206.x, PMID: 18507723

[22] Sheikh A Hurwitz B van Schayck CP McLean S Nurmatov U. Antibiotics versus placebo for acute bacterial conjunctivitis. Cochrane Database Syst Rev. (2012) 3:Cd001211. doi: 10.1002/14651858.CD001211.pub3, PMID: 22972049

[23] Jefferis J Perera R Everitt H van Weert H Rietveld R Glasziou P. Acute infective conjunctivitis in primary care: who needs antibiotics? An individual patient data meta-analysis. Br J Gen Pract. (2011) 61:e542–8. doi: 10.3399/bjgp11X593811, PMID: 22152728 PMC3162176

[24] Management of acute infective conjunctivitis. Management of acute infective conjunctivitis. Drug Ther Bull. (2011) 49:78–81. doi: 10.1136/dtb.2011.02.0043 21733975

[25] Tewfik GL Rodriguez-Aponte C Zhang K Ezzat B Suri P Chaudhry F. Outcomes and disposition of patients after case cancellation on day of surgery for reasons attributed to medical or anesthetic care: a retrospective cohort analysis. Anesth Analg. (2022) 135:845–54. doi: 10.1213/ANE.0000000000006156, PMID: 35913700

[26] Pattnaik S Dixit SK Bishnoi V. The burden of surgical cancellations: a quality improvement study on the importance of preoperative assessment. Cureus. (2022) 14:e21731. doi: 10.7759/cureus.21731 35251804 PMC8887625

[27] Mehran N Ojalvo I Myers JS Razeghinejad R Lee D Kolomeyer NN. Surgical cancellations in glaucoma practice: causes, delays, and effect on patient care and revenue. Ophthalmol Glaucoma. (2021) 4:427–32. doi: 10.1016/j.ogla.2020.12.006, PMID: 33338680

[28] Wongtangman K Azimaraghi O Freda J Ganz-Lord F Shamamian P Bastien A. Incidence and predictors of case cancellation within 24 h in patients scheduled for elective surgical procedures. J Clin Anesth. (2022) 83:110987. doi: 10.1016/j.jclinane.2022.110987, PMID: 36308990

[29] Umeno Y Ishikawa S Kudoh O Hayashida M. Effects of the multidisciplinary preoperative clinic on the incidence of elective surgery cancellation. J Med Syst. (2022) 46:95. doi: 10.1007/s10916-022-01883-3, PMID: 36374361 PMC9660193

[30] Ferschl MB Tung A Sweitzer B Huo D Glick DB. Preoperative clinic visits reduce operating room cancellations and delays. Anesthesiology. (2005) 103:855–9. doi: 10.1097/00000542-200510000-00025, PMID: 16192779

[31] van Klei WA Moons KG Rutten CL Schuurhuis A Knape JT Kalkman CJ. The effect of outpatient preoperative evaluation of hospital inpatients on cancellation of surgery and length of hospital stay. Anesth Analg. (2002) 94:644–9. doi: 10.1097/00000539-200203000-00030, PMID: 11867390

[32] Pollard JB Garnerin P Dalman RL. Use of outpatient preoperative evaluation to decrease length of stay for vascular surgery. Anesth Analg. (1997) 85:1307–11. doi: 10.1213/00000539-199712000-00023, PMID: 9390599

[33] Sato M Ida M Naito Y Kawaguchi M. The incidence and reasons for canceled surgical cases in an academic medical center: a retrospective analysis before and after the development of a preoperative anesthesia clinic. J Anesth. (2020) 34:892–7. doi: 10.1007/s00540-020-02841-4, PMID: 32793989

[34] Johnson BK James CW 3rd Ritchie G Morgan RR Jr McMillan HR. Evaluation of cost reduction measures at a state university medical center. J S C Med Assoc. (2014) 110:8–11. 27125004

